# Temperature Dependence of Electron Leakage Current in InGaN Blue Light-Emitting Diode Structures

**DOI:** 10.3390/nano12142405

**Published:** 2022-07-14

**Authors:** Chibuzo Onwukaeme, Bohae Lee, Han-Youl Ryu

**Affiliations:** Department of Physics, Inha University, 100 Inha-ro, Michuhol-gu, Incheon 22212, Korea; hyginusonwuka@gmail.com (C.O.); bbosea@inha.ac.kr (B.L.)

**Keywords:** electron blocking layer (EBL), GaN, light-emitting diode (LED), temperature dependence

## Abstract

We investigated the temperature dependence of the electron leakage current in the AlGaN electron-blocking layer (EBL) of an InGaN/GaN blue light-emitting diode (LED) structure at temperatures between 20 and 100 °C. The percentage of electron leakage current was experimentally determined by fitting the measured external quantum efficiency of an LED using the *ABC* recombination model. The electron leakage current decreased significantly as the temperature increased from 20 to 100 °C. The experiment obtained temperature-dependent electron leakage current was also found to agree well with the simulation results. This counter-intuitive temperature dependence of the electron leakage current resulted from an increase in potential barrier for electrons with increasing temperature due to the increased ionized acceptor concentration in the EBL with temperature. Moreover, the results obtained for the temperature-dependent electron leakage were consistent with the thermionic emission model. The results of the temperature dependence reported here are expected to provide insight into the thermal droop of GaN-based LEDs.

## 1. Introduction

The demand for light-emitting diodes (LEDs) for solid-state lighting display backlightings and automotive lamps continues to rise owing to their high efficiency and eco-friendliness [1,2,3,4]. The internal quantum efficiency (IQE) of InGaN/GaN blue LEDs can exceed 90% at relatively low current densities [5,6,7,8]. However, as the current density increases, the IQE of the InGaN blue LEDs decreases significantly [9,10,11,12]. In addition to this current droop, the temperature-dependent decrease in efficiency, known as thermal droop, has recently received increased attention [13,14,15,16,17,18]. It is important to understand the mechanisms behind the thermal droop of InGaN LEDs for use in high-power and temperature-stable applications. An increase in the nonradiative recombination rate and a decrease in the radiative recombination rate with increasing temperature can be one of the main causes of the thermal droop [16,17,18,19]. In our previous study, it was shown that the Shockley–Read–Hall (SRH) and Auger recombination coefficients increased with increasing temperature, while the radiative recombination coefficients decreased with temperature [19].

Electron leakage can have a significant influence on the thermal droop of InGaN LEDs because the electron leakage from a multiple-quantum-well (MQW) active region to a p-type layer has been attributed as one of the main reasons for current droop [20,21,22,23]. In typical InGaN blue LED structures, an electron-blocking layer (EBL) with a large energy bandgap material is inserted between MQWs and a p-GaN layer to suppress the electron leakage. There have been lots of studies on the design of EBL structures to decrease electron leakage current through effective blocking of electrons or improvement in hole injection [24,25,26,27,28,29]. One may expect that the electron leakage current increases with increasing temperature because thermionic emission generally increases with temperature [30,31]. However, most previous studies on EBL structures have investigated electron leakage characteristics only at room temperature, and few studies have investigated the temperature dependence of electron leakage characteristics of InGaN LEDs.

In this study, we investigated the electron leakage characteristics of InGaN blue LEDs in temperatures ranging from 20 to 100 °C both experimentally and theoretically. The electron leakage current can be determined by analyzing the IQE data of an InGaN LED. The IQE of LEDs is usually obtained by fitting the measured external quantum efficiency (EQE) as a function of current using the *ABC* carrier recombination model [10,32,33,34,35,36]. The measured EQE data can be fitted perfectly using the *ABC* model without carrier leakage effects at a relatively low current density. However, as the current density increases, the measured data become deviated from the IQE fitting curve based on the ideal *ABC* model. This deviation between the measured EQE data and the IQE fitting curve can be attributed to the electron leakage current [34,37].

We evaluated the electron leakage current using the deviation between the measured data and the fitting curves as the temperature varied. It will be shown later that the electron leakage current of a measured LED sample decreases with increasing temperature. To investigate the underlying mechanism for this temperature dependence, we performed numerical simulations of electron leakage at various temperatures. For the simulation study, a commercial software program, APSYS, was employed [38]. In addition, the measured and simulated temperature-dependent electron leakage characteristics were analyzed using a thermionic emission model at a hetero-interface. Based on the experimental and theoretical results of this study, the implications of temperature-dependent electron leakage for the thermal droop of InGaN blue LEDs will be discussed.

## 2. Experimental Results

### 2.1. Sample and Measurement

The LED samples in this study were grown on a c-plane sapphire substrate by metal–organic chemical vapor deposition. The layer structure consisted of a Si-doped *n*-GaN layer, an MQW active region, a 15 nm-thick p-type Mg-doped Al_0.15_Ga_0.85_N EBL, and a 150 nm-thick Mg-doped p-GaN layer. The MQW active layers were composed of five 3 nm-thick In_0.15_Ga_0.85_N QWs separated by 10 nm-thick GaN barriers. The LED chip was fabricated as a vertical injection structure with a chip dimension of 1 × 1 mm^2^. Next, the LED chip was mounted in a ceramic package as a type of surface-mount device. After that, the LED package was soldered on a copper block, and the temperature was controlled by a thermo-electric cooler (TEC).

**Figure 1:**
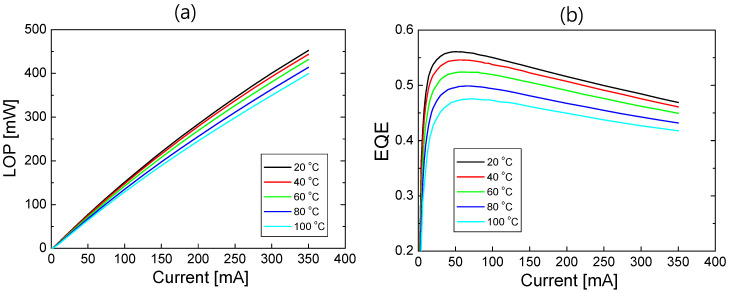
(**a**) Light output power (LOP) and (**b**) external quantum efficiency (EQE) of the measured LED sample as a function of injection current at temperatures of 20, 40, 60, 80, 100 °C.

The light output power (LOP) and spectral power distribution (SPD) of the packaged LED sample were measured as the TEC temperature varied from 20 to 100 °C. To minimize self-heating effects, the LED sample was driven under pulsed current injection with a pulse width of 1 ms and a duty cycle of 1%. As the temperature increased from 20 to 100 °C, the peak emission wavelength increased from 450 to 453 nm. The EQE of the sample was determined using the measured LOP and SPD. Figure 1 shows the LOP and EQE of the measured LED sample as a function of the injection current of up to 350 mA at temperatures of 20, 40, 60, 80, and 100 °C. The LOP and EQE decrease with increasing temperature. The peak EQE decreases from 0.544 to 0.464 as the temperature increases from 20 to 100 °C.

### 2.2. IQE Fitting and Leakage Current

In the *ABC* recombination model, the current *I* injected into the active region is composed of the recombination current *I_rec_* and leakage current *I_leak_* as expressed below:

(1) $$I=I_{rec}+I_{leak}=qV(An+Bn^{2}+Cn^{3})+I_{leak},$$

where *q* is the elementary charge, *V* is the active volume of the MQW layers, and *n* is the average carrier density of the MQWs. *I_rec_* includes the currents due to the following three carrier recombination processes in MQWs: SRH, radiative, and Auger recombination. The constants *A*, *B*, and *C* in Equation (1) are the coefficients for SRH, radiative, and Auger recombination, respectively. *I_leak_* represents the current due to electron leakage over the EBL to the p-GaN layer. The IQE of an LED is defined as follows:

(2) $$\eta =\frac{I_{rad}}{I}=\frac{Bn^{2}}{An+Bn^{2}+Cn^{3}+f_{leak}},$$

where *I_rad_* is the current due to radiative recombination, and *f_leak_* is equal to *I_leak_*/*qV*.

By fitting the measured EQE data using Equations (1) and (2), the EQE versus current relation, which is often referred to as the IQE curve, can be obtained. However, because of the leakage term, many fitting parameters are required and the IQE fitting becomes too complicated [34]. When there is no electron leakage (*I_leak_* = *f_leak_* = 0), the IQE fit curve can be reliably obtained using the peak IQE as a fit parameter without information of *ABC* coefficients [19,33]. We fitted the measured EQE data in Figure 1b using Equations (1) and (2) with *I_leak_* = 0. However, the IQE fitting over the entire current range of up to 350 mA was not successful owing to the existence of current leakage at high injection currents. When the fitting range was reduced to 100 mA, a nearly perfect fit was obtained.

**Figure 2:**
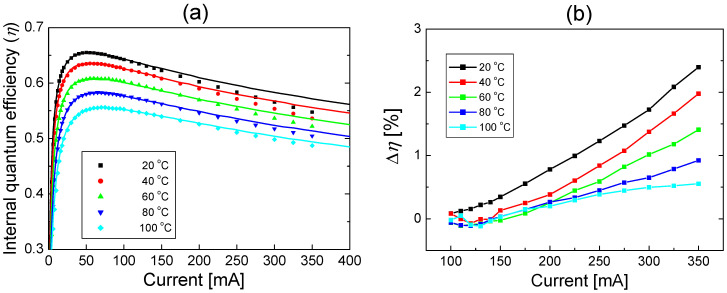
(**a**) Theoretical fit (lines) of the internal quantum efficiency (IQE) using *ABC* model without carrier leakage to the measured EQE data (solid dots) at temperatures of 20, 40, 60, 80, and 100 °C. (**b**) Difference in IQE (∆*η*) between the fit curve and the measured data at temperatures of 20, 40, 60, 80, and 100 °C when injection current is greater than 100 mA.

Figure 2a shows the IQE fit curves along with the measured data at 20, 40, 60, 80, and 100 °C. Here, the current range for the fitting was between 0 and 100 mA. As the temperature increases from 20 to 100 °C, the peak IQE decreases from 0.656 to 0.557. By comparing the peak EQE and peak IQE shown in Figure 1b and Figure 2a, the light extraction efficiency (LEE) that is defined as the ratio of EQE to IQE was determined to be ~0.83 at all temperatures. The measured IQE data shown in Figure 2a were obtained by dividing the EQE in Figure 1b by the LEE value. The temperature-dependent IQE fitting results without current leakage have been described in detail in Ref. [19].

In Figure 2a, a good agreement between the measured data and the IQE fit curves is observed for up to 100 mA at all temperatures, implying that the leakage current is almost negligible for injection currents <100 mA. In contrast, when the injection current is >100 mA, a deviation between the fit curves and measured data is observed at all temperatures, and the deviation increases as the injection current increases. The experimental IQE was lower than the fitted IQE, which can be attributed to the additional decrease in IQE owing to the leakage current. Since *f_leak_* corresponds to the deviation from the *ABC* model fit, it is implicitly assumed that *f_leak_* has fourth-order or higher dependence on carrier density. However, *f_leak_* may contain a third-order dependence term as presented in Ref. [37]. In that case, it is difficult to extract the leakage component from the *Cn*^3^ term in our model. In this study, we restrict our attention to the conventional *ABC* model, where the coefficient *C* represents the Auger recombination only.

Figure 2b shows the difference in IQE (∆*η*) between the fit curve and the measured data when the injection current is greater than 100 mA. The difference in the IQE values increases as the injection current increases, implying that the leakage current increases with increasing injection current. In addition, the difference in the IQE values decreases as the temperature increases, implying that the leakage current decreases with increasing temperature. The leakage current can be obtained using Equations (1) and (2). When the difference between the measured and fitted IQE is small, the leakage current can be simply expressed as below:

(3) $$I_{leak}=\frac{\Delta \eta }{\eta }I.$$

**Figure 3:**
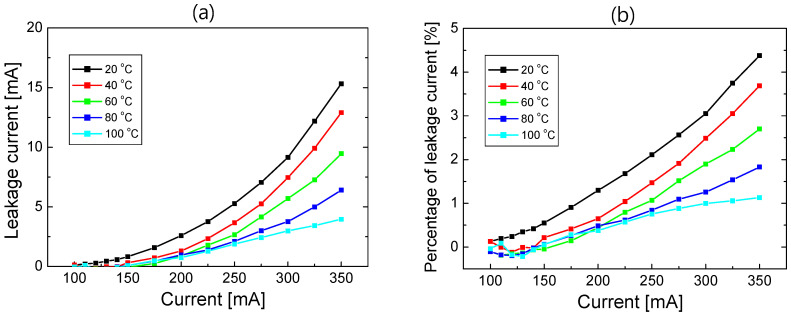
(**a**) Leakage current as a function of injection current and (**b**) the percentage of electron leakage current (*P_leak_*) as a function of injection current at temperatures of 20, 40, 60, 80, and 100 °C.

In Figure 3a, the leakage current in Equation (3) is plotted as a function of the injection current at each temperature. The leakage current increases significantly as the injection current increases or the temperature decreases. This temperature dependence of the electron leakage current may seem to be counterintuitive, considering that the probability of thermionic emission increases with increasing temperature because of the increased thermal energy of electrons. The reduced electron leakage with increasing temperature can be attributed to the increase in hole concentration and improvement in hole transport with temperature. The mechanism behind this temperature dependence will be investigated in detail in a subsequent section. Figure 3b shows the percentage of leakage current (*P_leak_*), defined as the ratio of the leakage current to the injected current, with the varying injection current and temperature. *P_leak_* increases steadily as the current increases and the temperature decreases. At 350 mA, for example, *P_leak_* decreases from 4.38% to 1.13% as the temperature increases from 20 to 100 °C.

## 3. Simulation Results

### 3.1. Simulation Model

The temperature dependence of the electron leakage characteristics was investigated by conducting numerical simulations using the APSYS program. The transport model of the program, which plays an important role in carrier leakage, includes the drift and diffusion equations of electrons and holes, Fermi statistics, and thermionic emission at hetero-interfaces. The electron leakage characteristics can be strongly influenced by the built-in polarization field and conduction band offset [39]. The built-in polarization fields induced by spontaneous and piezoelectric polarization were included at the InGaN/GaN, AlGaN/GaN, and InGaN/AlGaN hetero-interfaces using the model described in Ref. [40], assuming a 50% compensation for the polarization fields. The conduction band offset of the InGaN/GaN and AlGaN/GaN interfaces was set to 0.75. It will be shown later that the experimentally obtained temperature-dependent electron leakage current agrees well with the simulation results for these parameter values. The polarization compensation factor and the conduction band offset were assumed to be independent of the temperature from 20 to 100 °C.

The simulated LED structure is basically the same as that of the fabricated structure. For the doping concentrations of the LED layer structures, the MQW region was left undoped and the n-GaN layer was doped by Si with a doping concentration of 5 × 10^18^ cm^−3^. The Mg doping concentration of the p-AlGaN EBL and p-GaN layers was 5 × 10^18^ cm^−3^. In the p-type doped layers, incomplete ionization of Mg acceptors was included such that the acceptor ionization energy in AlGaN scaled linearly from 170 meV (GaN) to 470 meV (AlN) [41,42]. The thermal activation model for Mg acceptors was also included. The field- and temperature-dependent mobility model described in Refs. [43,44,45] was used for the mobility of electrons, which resulted in an electron mobility of ~500 cm^2^/Vs for an electron concentration of 1 × 10^18^ cm^−3^. The hole mobilities in AlGaN, GaN, and InGaN layers were assumed to be 15, 10, and 5 cm^2^/Vs, respectively [45].

In our previous study [19], the temperature dependence of *ABC* coefficients was obtained as follows: *A* increased from 7.6 × 10^6^ to 9.8 × 10^6^ s^−1^, *B* decreased from 0.66 × 10^−10^ to 0.49 × 10^−10^ cm^3^/s, and *C* increased from 2.07 × 10^−30^ to 2.72 × 10^−30^ cm^6^/s, as the temperature was increased from 20 to 100 °C. These temperature dependences of *ABC* coefficients were also included in the simulation for the electron leakage current of this study.

### 3.2. Simulation of Electron Leakage Current

**Figure 4:**
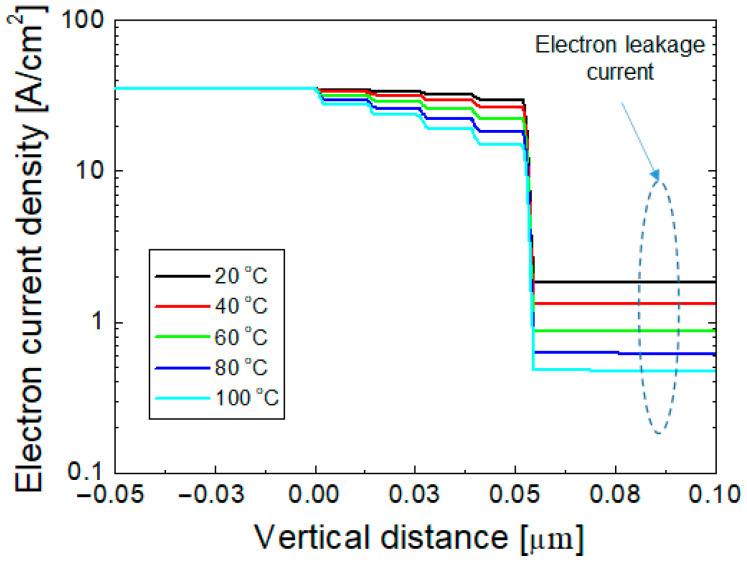
Simulated electron current density as a function of vertical position at 20, 40, 60, 80, and 100 °C when the injection current is 350 mA.

Figure 4 shows the vertical component of the electron current density as a function of the vertical position at 20, 40, 60, 80, and 100 °C with an injection current of 350 mA. The electron current density at the n-GaN layer is 35 A/cm^2^ for all temperatures, which decreases gradually at the MQWs owing to the recombination of electrons with holes. Because the overflow of electron current across the EBL is considered as the electron leakage current, the electron current at the p-GaN layer corresponds to the electron leakage current, as indicated in Figure 4. It can be seen that the electron leakage current density decreases as the temperature increases. This temperature dependence is consistent with the experimental results shown in Figure 2 and Figure 3. As the temperature of an LED increases from 20 to 100 °C, the leakage current density decreases from 1.82 to 0.47 A/cm^2^.

**Figure 5:**
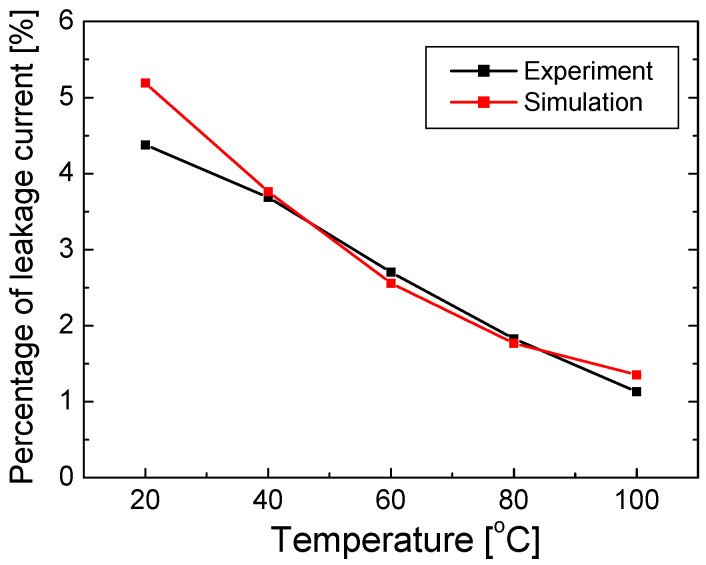
Simulated and experimented *P_leak_* values as a function of temperature ranging from 20 to 100 °C.

In the simulations, *P_leak_* could be obtained by dividing the electron current density of the p-GaN layer by that of the n-GaN layer. In Figure 5, the simulated *P_leak_* is compared to *P_leak_* shown in Figure 3b, which was obtained by fitting the measured data, as the temperature varies from 20 to 100 °C. Here, the injection current was 350 mA. A reasonably good agreement is found between the simulated and experimental *P_leak_* values. For the polarization compensation factor of 50% and the conduction band offset of 0.75, the simulation successfully explained the experimentally obtained temperature-dependent electron leakage current.

### 3.3. Analysis Uising Thermionic Model

**Figure 6:**
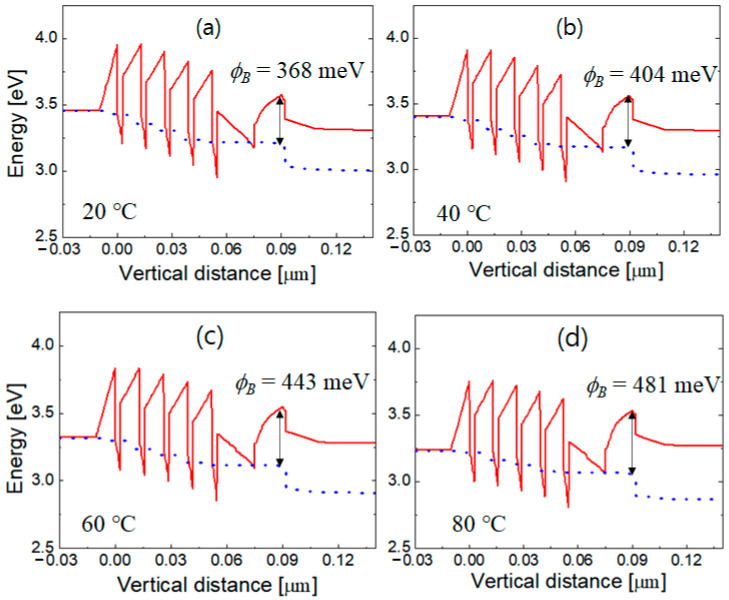
Conduction band diagrams at (**a**) 20, (**b**) 40, (**c**) 60, and (**d**) 80 °C when the injection current is 350 mA. The solid and dotted lines indicate the conduction band edge and electron quasi-Fermi energy level, respectively. The effective electron potential ($\phi_{B}$) at 20, 40, 60, and 80 °C is 368, 404, 443, and 481 meV, respectively. The origin of the horizontal axis corresponds to the position of the effective potential barrier height.

Electron leakage through the EBL is expected to be mainly governed by the thermionic emission process. The leakage current density caused by thermionic emission at a hetero-interface with a potential barrier $\phi_{B}$ is expressed as [46]

(4) $$J=A^{*}T^{2}\exp(-\frac{q\phi_{B}}{kT}),$$

where A^*^ is the effective Richardson constant, *q* is the elementary charge, *k* is the Boltzmann constant, and *T* is the absolute temperature. Figure 6 shows the simulated energy band diagrams at 20, 40, 60, and 80 °C with an injection current of 350 mA. The solid and dotted lines indicate the conduction band edge and the electron quasi-Fermi energy level, respectively. The effective electron potential barrier $\phi_{B}$ corresponds to the difference between the conduction band edge and the electron quasi-Fermi energy level at the p-side edge of the AlGaN EBL. In Figure 6, the origin of the horizontal axis corresponds to the position of the effective potential barrier height. It can be seen that $\phi_{B}$ increases as the temperature increases. At 20, 40, 60, and 80 °C, the corresponding $\phi_{B}$ values are 368, 404, 443, and 481 meV, respectively.

**Figure 7:**
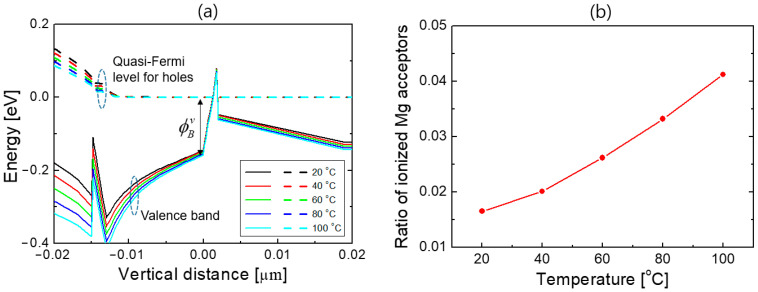
(**a**) Valence band diagrams at 20, 40, 60, and 80 °C when the injection current is 350 mA. The solid and dotted lines indicate the valence band edge and hole quasi-Fermi energy level, respectively. The origin of the horizontal axis corresponds to the position of the maximum effective potential barrier height. $\phi_{B}^{v}$ represents the difference between the valence band edge and the hole quasi-Fermi energy level at the origin. (**b**) Ratio of ionized Mg acceptors as a function of temperature.

As the temperature increases, the hole concentration in the EBL increases owing to the thermal activation of the Mg acceptors. At room temperature, the ionization ratio of Mg in AlGaN is very low, only a few percent or even less than 1%, because of the large acceptor ionization energy [47]. The ionization ratio of Mg can be increased significantly with increasing temperature, resulting in an increase in the negatively charged Mg acceptor ions with temperature. The increase in the concentration of ionized Mg acceptors increases the Coulomb repulsion of electrons, which appears with the increase in the effective potential barrier for electrons. Figure 7a shows the valence band diagrams at 20, 40, 60, and 80 °C when an injection current is 350 mA. $\phi_{B}^{v}$ denotes the difference between the valence band edge and the hole quasi-Fermi energy level at the origin that corresponds to the effective potential barrier for electrons. It increased slightly from 146 to 158 meV as the temperature increased from 20 to 100 °C.

For an acceptor doping concentration of *N_a_*, the ratio of ionized accepter concentration $N_{a}^{-}$ to *N_a_* is given by [47,48]

(5) $$\frac{N_{a}^{-}}{N_{a}}=1-\frac{1}{1+g_{a}^{-1}\exp[(E_{a}-E_{Fp})/kT]}$$

where *E_Fp_*, *E_a_*, *k*, and *T* are the hole quasi-Fermi energy level, acceptor ionization energy, Boltzmann constant, and the absolute temperature, respectively. *g_a_* is called a degeneracy factor, which is normally taken as 4 for acceptors. For the Al_0.15_Ga_0.85_N EBL, *E_a_* − *E_v_* = 215 meV [41,42], where *E_v_* is the valence band edge, and $\phi_{B}^{v}$ equals to *E_Fp_* − *E_v_*. Therefore, the ratio of the ionized Mg acceptors in Equation (5) can be calculated as the temperature varies using $\phi_{B}^{v}$ values in Figure 7a. Figure 7b plots the ratio of ionized Mg acceptors as a function of temperature. It increased from 1.6% to 4.1% as the temperature increased from 20 to 100 °C, which reveals that the ionization ratio of Mg acceptor in the AlGaN EBL can be increased significantly as the temperature increases. Consequently, the electron potential barrier increases as the temperature increases, which helps to suppress electron leakage. The thermionic model in Equation (4) implies that the increase in $\phi_{B}$ with increasing temperature is the main reason for the decreased electron leakage current with temperature, despite the increase in the thermal energy of electrons with temperature.

Using Equation (4), the ratio of the electron leakage current density between temperatures *T*_1_ and *T*_2_ can be expressed as follows:

(6) $$\frac{J(T_{2})}{J(T_{1})}={\left(\frac{T_{2}}{T_{1}}\right)}^{2}\exp[\frac{q}{k}(\frac{\phi_{B}(T_{1})}{T_{1}}-\frac{\phi_{B}(T_{2})}{T_{2}})].$$

**Figure 8:**
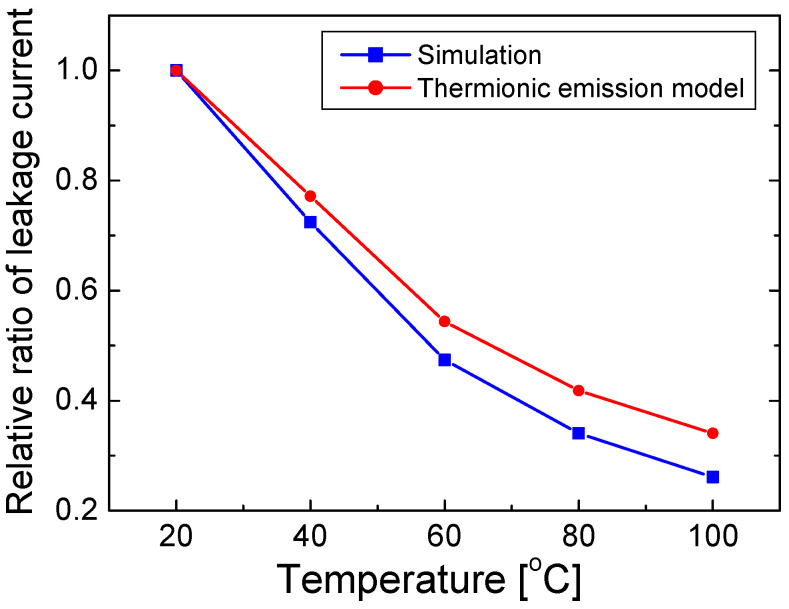
Relative ratios of leakage current (*R_leak_*) obtained through simulation results in Figure 5, and *R_leak_* values obtained using the thermionic emission model in Equation (6) are plotted as a function of temperature.

Using Equation (6) and the temperature-dependent $\phi_{B}$ values in Figure 6, the relative ratio of leakage current (*R_leak_*), which is defined as *P_leak_* at temperature *T* normalized by *P_leak_* at 20 °C, is calculated as a function of temperature. Figure 8 shows the calculated *R_leak_* value obtained using Equation (6) along with the *R_leak_* value obtained from the simulation result (Figure 5) as a function of temperature varying from 20 to 100 °C.

In Figure 8, the calculated and simulated *R_leak_* values are shown as red and blue lines, respectively. The values of *R_leak_* decrease with increasing temperature, as expected from the result in Figure 5. As the temperature increases, $\phi_{B}$ increases such that $\phi_{B}$(*T*_2_)/*T*_2_ is greater than $\phi_{B}$(*T*_1_)/*T*_1_ for *T*_2_ > *T*_1_, resulting in a decrease in *R_leak_* values with increasing temperature, despite the (*T*_2_/*T*_1_)^2^ factor in Equation (5). The calculated *R_leak_* value was slightly higher than the simulated value. The relative difference was 5–25% as the temperature varied. Because the calculation of *R_leak_* was based on the thermionic model, the small difference between the calculated and simulated values implies that the temperature-dependent electron leakage can be explained mainly by the thermionic model.

Based on the experimental and theoretical results of this study, it was revealed that the electron leakage current decreased with increasing temperature. This strongly suggests that electron leakage should be excluded as a possible origin of the thermal droop of InGaN-based blue LEDs, and the increase in the nonradiative recombination rate and a decrease in the radiative recombination rate with increasing temperature can be regarded as the main causes of the thermal droop.

## 4. Conclusions

In this study, we investigated the temperature dependence of the electron leakage current in InGaN blue LED structures, both experimentally and theoretically. The percentage of electron leakage current was evaluated using the deviation between the measured efficiency data and an IQE fit curve based on the *ABC* model. Contrary to common expectations, the electron leakage current decreased significantly as the temperature increased from 20 to 100 °C. The decrease in the electron leakage current with increasing temperature was confirmed by conducting numerical simulations, which was also found to be consistent with theoretical analyses based on the thermionic emission model. The decrease in electron leakage current was attributed to an increase in potential barrier for electrons as the temperature increased due to the increase in ionized Mg acceptor concentration in the AlGaN EBL with temperature. The results of this study imply that electron leakage can be excluded as a mechanism behind the thermal droop of InGaN blue LEDs.

## Data Availability

The data supporting the findings of this paper are available from the corresponding authors upon reasonable request.
